# Oxygen-Dependent Patient with Antisynthetase Syndrome Associated Interstitial Lung Disease Responds Promptly to Rituximab with Rapid Pulmonary Function Improvement

**DOI:** 10.31138/mjr.28.3.153

**Published:** 2017-09-29

**Authors:** Robert Chao, Mukund Das, Cecil Philip, Petros Efthimiou

**Affiliations:** Department of Medicine, Weill Cornell Medicine/New York-Presbyterian Brooklyn Methodist Hospital, Brooklyn, NY, USA

**Keywords:** Antisynthetase syndrome, myositis, interstitial lung disease, rituximab

## Abstract

Antisynthetase syndrome (anti-SS) is a rare systemic autoimmune disorder characterized by myositis, Raynaud’s phenomenon, fever, interstitial lung disease (ILD), polyarthralgia, and presence of antibodies against tRNA synthetase, especially anti-Jo-1. Rarely, anti-SS can present as isolated ILD, with clinical features very similar to atypical pneumonia, making diagnosis extremely challenging. We report a patient originally diagnosed with atypical pneumonia, requiring oxygen supplementation, who failed treatment with antibiotics. Radiological findings were suspicious for ILD and a comprehensive rheumatological work-up revealed the diagnosis of anti-SS associated ILD. Prompt treatment was initiated with steroids and rituximab. Follow up pulmonary function tests showed an improvement in her diffusing capacity of the lung for carbon monoxide and forced vital capacity allowing her to resume her daily life without supplemental oxygen.

## INTRODUCTION

Inflammatory myopathies comprise a diverse group of autoimmune disorders and pulmonary involvement is one of the most common extra-muscular manifestations. Antisynthetase syndrome (anti-SS) is a major subgroup of inflammatory myopathies characterized by interstitial lung disease (ILD), inflammatory myositis, fevers, mechanic’s hands, Raynaud’s phenomenon, polyarthralgia, and presence of antibodies against amino acyl-transferase RNA synthetase. The incidence of anti-SS is unknown; however, the incidence of inflammatory myopathies ranges between 2 to 10 cases per million per year and antisynthetase autoantibodies are found in 25 – 40% of cases.^1,5^ Anti-SS primarily manifests between the ages of 43 to 60, with a female predominance.^2^ The pathogenic mechanism by which antibodies against amino acyl-transferase RNA synthetase trigger autoimmune response is very poorly understood. Of this group of antibodies, anti-Jo-1 is the most common and myositis-specific autoantibody. Studies have shown a direct correlation of anti-Jo-1 antibody levels with severity of muscle and lung involvement in anti-SS.^3^ Other much less frequent autoantibodies include anti-PL12, anti-PL7, and anti-OJ.

Although clinical presentation varies, most anti-SS patients present with ILD, inflammatory myositis and polyarthritis. Myositis is seen in more than 90% of patients; typically presenting with profound weakness in the proximal muscle groups, however, in rare instances, patients may be asymptomatic and only have a transient rise in creatine kinase. Non-erosive arthritis is typically observed but joint erosion and destruction can also occur.^4^ ILD has been reported in about 90% of patients with anti-SS, often presenting with acute, subacute, or insidious onset of exertional dyspnea.^4^ Physical exam findings of inspiratory crackles and chest radiography findings of interstitial changes are not sensitive and can often miss early ILD. If anti-SS is suspected, a high resolution computed tomography of the chest showing ground glass opacities can be confirmatory.^4^ Despite the medical need for treatment options for anti-SS associated ILD, data remains scarce, limited to few case reports or small case series.

Treatment with high dose steroids may not be adequate for patients with anti-SS who present with oxygen dependent ILD. Due to anti-Jo-1 antibody’s role in the pathophysiology of anti-SS, more clinicians are utilizing the B-cell depleting monoclonal anti-CD20 antibody, rituximab as a treatment option. Recent case reports are limited but over the past 20 years there is growing evidence for significant improvement of ILD in anti-SS patients receiving rituximab.^6^ There have been documented improvements in both the diffusing capacity of the lung for carbon monoxide (D_LC_^O^) and forced vital capacity (FVC) in such patients.^7^ We present a patient with debilitating anti-SS associated ILD who responded rapidly to rituximab with documented improvement in pulmonary function.

## CASE DESCRIPTION

**Figure 1.**
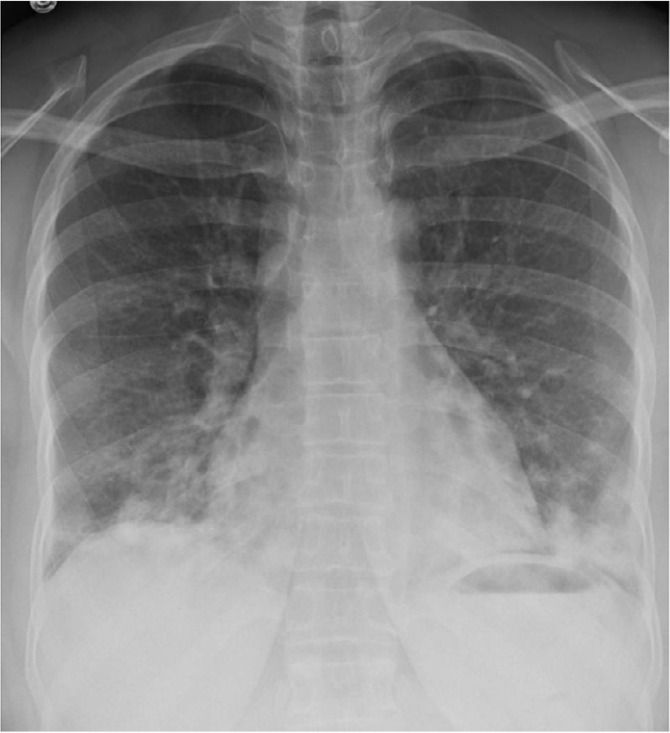


**Figure 2.**
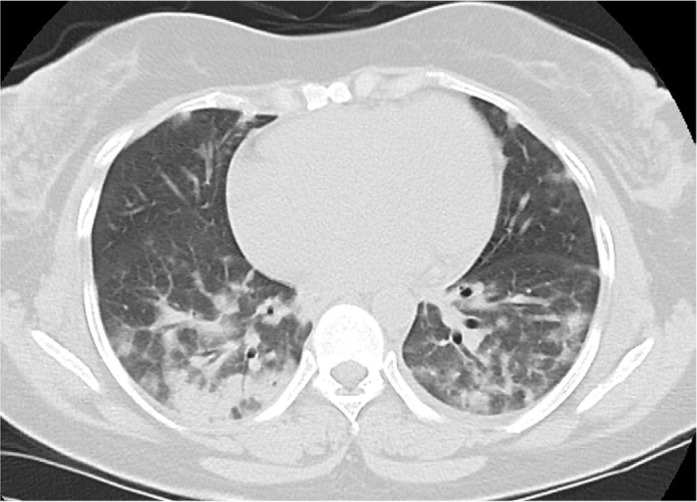


A 55-year-old female with no medical history presented with a dry cough for one month associated with decreased exercise tolerance and dyspnea on exertion in addition to bilateral pleuritic chest pain. At baseline, she had no difficulty walking long distances and was able to carry out activities of daily living with no difficulty. However, within the past month, she was only able to walk half a block before becoming short of breath. She denied any recent respiratory illnesses, joint swelling, or muscle weakness. Initial vital signs were temperature of 98°F, blood pressure of 116/76 mmHg, respiratory rate of 18 breaths per minute, and oxygen saturation of 95% on room air. Pulmonary examination revealed bibasilar “Velcro-like” dry inspiratory crackles. Cardiovascular, abdominal, and neurological examinations were unremarkable. Laboratory tests revealed microcytic anemia and no leukocytosis. Routine biochemistry and liver function tests were normal. Her erythrocyte sedimentation rate was 26 (0–15 mm/hr), C-reactive protein 35.9 (0–3 mg/dL), and creatine kinase level were elevated at 464 (21–215 units/L). Urinalysis and hepatitis serology were normal. Chest radiograph showed bibasilar airspace infiltrates more pronounced on the left (**Figure 1**). *Legionella* antigen, *Mycoplasma* antigen, blood culture, and QuantiFERON Gold were all negative. She was treated for community acquired pneumonia with ceftriaxone and azithromycin but during her hospital course, she continued to desaturate to 80% upon exertion. Computed tomography of the chest showed extensive bilateral lower lobe opacities along with scattered ground glass opacities and peri-bronchial consolidations (**Figure 2**). Her gender, age, and radiological findings suspicious for ILD prompted a rheumatology workup, which was positive for rheumatoid factor, antinuclear antibody, cyclic citrullinated peptide, and anti-Jo-1 antibody. A subsequent right quadriceps muscle biopsy revealed active, mild myopathy with focal subtle endomysial fibrosis suggestive of immune-mediated etiology.

**Figure 3.**
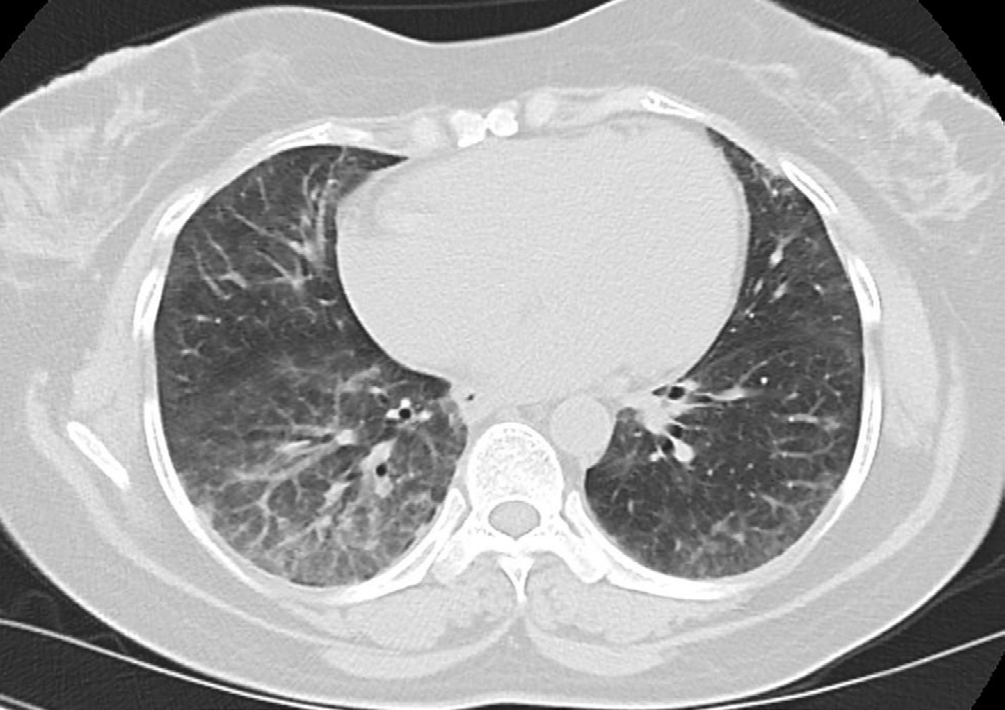


She was diagnosed with antisynthetase syndrome presenting primarily with ILD. The patient was started on intravenous pulse dose steroids (1gm methylpredniso-lone) along with mycophenolate mofetil (MMF) resulting in significant improvement in her clinical symptoms. She was discharged home on tapering steroids, MMF, and oxygen supplementation with close follow up. Outpatient treatment with rituximab allowed her to be weaned off oxygen supplementation and eventually regain her baseline level of activity. A follow up computed tomography of the chest showed interval decrease in her bibasilar infiltrates (**Figure 3**). Her initial and 5-month post-treatment pulmonary function tests showed an improvement in D_LCO_ and FVC (**Table 1**).

**Table 1:** Antisynthetase Syndrome Pulmonary Function Analysis.

**Pulmonary Function Test**	**Initial**	**After Rituximab**
FEV1	38%	53%
FEV1/FVC	74%	74%
FVC	31%	40%
TLC	3.36 L	2.49 L
D_LCO_	19%	32%

FEV1 = forced expiratory volume in 1 second

FEV1/FVC = forced expiratory volume in 1 second/forced vital capacity

FVC = forced vital capacity

TLC = total lung capacity

D_LCO_ = diffusing capacity of the lung for carbon monoxide

The initial and post-rituximab pulmonary function tests showing marked improvement in patient’s diffusing capacity of the lung for carbon monoxide and forced vital capacity

## DISCUSSION

Antisynthetase syndrome, initially described in 1990 as a triad of polymyositis, ILD and positive autoantibodies is a rare condition and even fewer cases have been identified with ILD as the sole presentation. Presence of arthritis, mechanic’s hands, and Raynaud’s phenomenon are not necessary but support the diagnosis. The prognosis of anti-SS is primarily based on the degree of ILD and its response to treatment. Anti-Jo-1 antibody levels correlate directly with severity of muscle and lung involvement in anti-SS.^3^

Treatment of anti-SS associated ILD is still not established and current therapy is based on starting corticosteroids as initial treatment. Immunosuppressive medications such as cyclophosphamide, azathioprine, mycophenolate mofetil, cyclosporine and tacrolimus are also routinely used.^8^ Given anti-Jo-1 antibody’s direct correlation with anti-SS associated ILD, rituximab’s role as an anti-CD20 B-cell depleting monoclonal antibody has grown. Anti-SS patients treated with rituximab have shown improvement in ILD and its associated symptoms.^9^ Similar to our patient, previous studies have shown an improvement in pulmonary function, specifically D_LCO_ and FVC in anti-SS patients after treatment with rituximab.^7^ In a recent study, not only did rituximab show improvement in pulmonary function but also led to a decrease in total creatine kinase levels, decreased steroid dosage, and decreased usage of immunosuppressive medications.^10^ Interestingly, pulmonary function improvement may also be due to muscular strength reinforcement rather than just improvement of ILD itself.^10^ During treatment, adverse effects of rituximab were mainly limited to infections, but not severe enough to require hospitalization.^10^ Early diagnosis of antisynthetase syndrome along with prompt initiation of the correct treatment regimen is critical to preventing disease progression. Recent research is limited to case reports and retrospective studies; nevertheless, they indicate treatment with rituximab have shown great efficacy in improving pulmonary function. We hope this supports the need for prospective clinical trials focusing exclusively on antisynthetase patients to test for the efficacy of rituximab and its long-term effects on interstitial lung disease.
